# Liquid Biopsy for the Detection of Resistance Mechanisms in NSCLC: Comparison of Different Blood Biomarkers

**DOI:** 10.3390/jcm8070998

**Published:** 2019-07-09

**Authors:** Luigi Pasini, Paola Ulivi

**Affiliations:** Biosciences Laboratory, Istituto Scientifico Romagnolo per lo Studio e la Cura dei Tumori (IRST) IRCCS, 47014 Meldola, Italy

**Keywords:** liquid biopsy, diagnostic biomarkers, targeted therapy, acquired resistance, lung cancer

## Abstract

The use of targeted agents and immunotherapy for the treatment of advanced non-small-cell lung cancer (NSCLC) has made it mandatory to characterize tumor tissue for patient selection. Moreover, the development of agents that are active against specific resistance mechanisms arising during treatment make it equally important to characterize the tumor tissue at progression by performing tissue re-biopsy. Given that tumor tissue is not always available for molecular characterization due to the paucity of diagnostic specimens or problems relating to the carrying out of invasive procedures, the use of liquid biopsy represents a valid approach to overcoming these difficulties. The most common material used for liquid biopsy in this setting is plasma-derived cell free DNA (cfDNA), which originates from cells undergoing apoptosis or necrosis. However, other sources of tumor material can be considered, such as extracellular vesicle (EV)-derived nucleic acids, which are actively secreted from living cells and closely correspond to tumor dynamics. In this review, we discuss the role of liquid biopsy in the therapeutic management of NSCLC with particular regard to targeted therapy and immunotherapy, and analyze the pros and cons of the different types of samples used in this context.

## 1. Introduction

The use of non-invasive methods to characterize tumors is a valuable tool for the management and assessment of cancer patients, e.g., at diagnosis, for the prediction of prognosis and response to therapy, and in the monitoring of response to treatment. The introduction of targeted therapy into clinical practice has made it mandatory to characterize tumor tissue for patient selection, increasing the need for biological material for molecular analysis. In patients with advanced non-small-cell lung cancer (NSCLC) for whom diagnosis is normally made on small biopsies or cytology specimens, the frequent paucity of available tumor tissue is a serious problem. Moreover, if the biological sample available derives from a bone biopsy, there is a higher risk of bad quality material that can compromise the molecular analysis. In addition, the tumor may be heterogeneous and a biopsy performed at a specific point may consequently not represent the overall molecular landscape of the cancer. Liquid biopsy represents an optimal strategy to overcome such problems. Another important use of liquid biopsy is to monitor response to treatment and the onset of resistance mechanisms. Given that tumor re-biopsy is an invasive procedure and that not all patients are amenable to surgery, the only remaining option is to characterize tumor alterations by liquid biopsy. Molecular analysis of liquid biopsy normally takes less time than that of tumor tissue, which involves several steps and evaluations performed by a pathologist, with a consequent lengthening of the turnaround time. 

As the amount of tumor material present in biological fluids is very limited, it is crucial to use highly sensitive methodologies to avoid false-negative results and guarantee the best treatment options for patients. Although different types of biological samples are used for liquid biopsies, cell free DNA (cfDNA) is currently the most widely used material in clinical practice.

In this review, we report on the use of liquid biopsy in NSCLC treatment, in particular, targeted therapy and immunotherapy, and focus on the different types of biological samples used for molecular analyses, weighing the pros and cons of each approach.

## 2. Targeted Therapy in NSCLC

**Figure 1:**
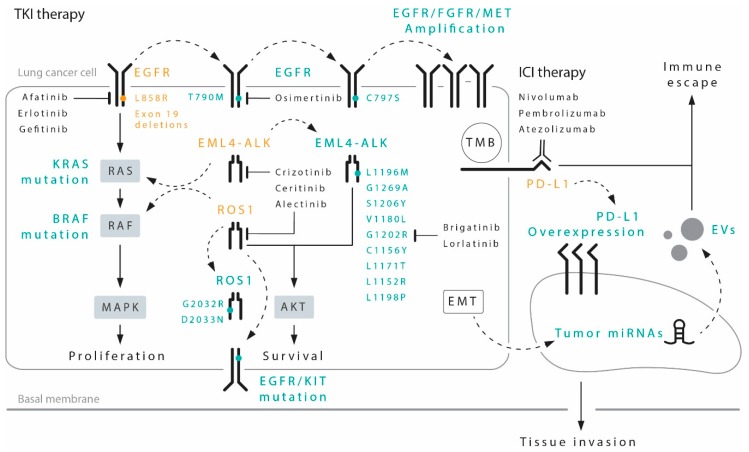
Most relevant targeted therapies and immune checkpoint inhibitor (ICIs) in non-small-cell lung cancer (NSCLC), with the principal targets highlighted (in orange) and the most recurrent resistance mechanisms (in green) potentially detectable by liquid biopsy. EGFR, epidermal growth factor receptor; KIT, c-Kit proto-oncogene receptor tyrosine kinase; MET, c-Met proto-oncogene; EML4-ALK, echinoderm microtubule associated protein-like 4-anaplastic lymphoma kinase; ROS1, ROS1 proto-oncogene receptor tyrosine kinase; EV, extracellular vesicles.

Tyrosine kinase inhibitors (TKIs) represent the treatment of choice for subgroups of patients carrying specific gene alterations (Figure 1). In particular, patients harboring mutations of the epidermal growth factor receptor (EGFR), as well as rearrangements of the anaplastic lymphoma kinase (ALK) or the ROS proto-oncogene 1 (ROS1), have significantly improved clinical outcomes when treated with TKI therapy.

Specific driver mutations can identify patients that are sensitive to certain target inhibitors, whereas the expression of the programmed death-ligand 1 (PD-L1) and elevated tumor mutational burden (TMB), may help define the patients with higher probability to respond to immune checkpoint inhibitors (ICIs). A series of FDA-approved tyrosine kinase inhibitor (TKIs) and ICIs are now available for the first-line treatment of NSCLC. Acquired resistance, however, can promote downstream activation (straight arrowed lines) or bypass (dotted arrows) of signaling pathways, via secondary mutations in the same target gene, as well as parallel mutations or amplification of membrane receptors (e.g., EGFR, FGFR, MET, KIT), and transcription of tumor-specific miRNAs. Emergence of epithelial-to-mesenchymal transition (EMT) is a common trait of advanced-cancer clones that have developed pharmacological resistance. All these mechanisms of pharmacological resistance are potentially detectable via liquid biopsy. 

### 2.1. Anti-Epidermal Growth Factor Receptor 

A new era in NSCLC treatment started in 2004 when it was discovered that mutations of *EGFR* gene are predictive of sensitivity to therapy with anti-EGFR TKIs [1,2]. Following the disappointing results of the IDEAL-1 and IDEAL-2 trials for gefitinib [3,4], and the TALENT and TRIBUTE trials for erlotinib [5,6], both drugs continued to produce contrasting results until the success of several studies in which EGFR-TKIs were compared to chemotherapy in patients with activating *EGFR* mutations [7,8,9]. In this setting, both drugs were shown to prolong progression-free survival (PFS) but not overall survival (OS) compared with platinum-doublet chemotherapy (median PFS of 9.2–13.1 months vs. 4.6–6.3 months, respectively). 

Afatinib, a second-generation EGFR-TKI that irreversibly blocks EGFR and other ErbB-family receptors, demonstrated a benefit in PFS with respect to chemotherapy in two phase III trials, (LUX-Lung 3 and LUX-Lung 6) [10,11]. It also showed a significant advantage in terms of OS in patients carrying EGFR exon 19 deletions [12]. Subsequently, osimertinib, a third-generation EGFR-TKI developed for the second-line treatment of patients progressing on first- or second-generation TKIs and developing an EGFR-T790M mutation [13], prolonged PFS with respect to gefitinib and erlotinib in previously untreated EGFR-mutated patients. It has since been approved for use in a first-line setting [14].

However, although a clinical benefit is indubitably observed with the use of these drugs, resistance mechanisms inevitably appear after around 12 months of treatment [7,8,9,10,11]. The most common resistance mechanism to first- and second-generation TKIs is the gatekeeper T790M mutation occurring at exon 20 of *EGFR* (Figure 1) in about 50–70% of cases. It has been seen that patients developing this alteration remain sensitive to osimertinib, thus explaining why this drug was approved for second-line therapy in this subgroup [7]. This has led to the need for tumor characterization after progression on first- or second-line TKIs, which poses the problem of having sufficient tumor tissue available for molecular analysis. As tumor re-biopsy is not always possible due to tumor localization or patients’ health conditions, liquid biopsy has become a key element in the determination of resistance mutations. 

In contrast to first- and second-generation TKIs, no predominant acquired resistant mechanisms have been observed for osimertinib in the first-line setting, for which very few data are still available. A number of in vitro studies and case reports have shown that secondary EGFR mutations [15,16], activation of AXL [17] and ERK [18] signaling, and small cell transformation [19] may emerge as acquired resistance mechanisms in this setting. More data are available on resistance mechanisms to second-line osimertinib in T790M-positive patients. In these patients, C797S mutation is observed in around 20–40% of cases, while amplification of the c-Met proto-oncogene (*MET*) in 14% of cases; at lower frequency, transformation into small-cell phenotype, amplification of the erb-B2 receptor tyrosine kinase 2 (*HER2*) or the fibroblast growth factor receptor 1 (*FGFR1*) genes, and mutations in the b-raf proto-oncogene, serine/threonine kinase (*BRAF*) is also observed [20,21].

### 2.2. Anti-Anaplastic Lymphoma Kinase

In 2007, aberrant gene fusion of the *echinoderm microtubule associated protein-like 4 (EML4)* with *ALK* was documented in NSCLC [22]. Genetic rearrangement of *ALK* is present in about 3–7% of NSCLC patients who are usually young, non-smokers and have tumors with adenocarcinoma (ADC) histology [23]. The first drug developed against this alteration was crizotinib, an oral ATP-competitive selective inhibitor of the *ALK*, *MET*, and *ROS1* tyrosine kinase. Two phase III trials, PROFILE 1007 [24] and PROFILE 1014 [25] showed a significant advantage of crizotinib over chemotherapy in first- and second-line settings, respectively. Based on these results, the drug was approved for treatment of patients with advanced ALK-rearranged tumors, becoming the treatment of choice in untreated NSCLC patients carrying this driver alteration. However, after about 12 months’ treatment the majority of patients progressed by developing both ALK-dependent and -independent resistance mechanisms. The most common secondary *ALK* mutations are L1196M and G1269A [26,27,28], both of which interfere with crizotinib binding. Other mutant variants that reduce the ATP-binding affinity of crizotinib include S1206Y, V1180L and G1202R, whereas other secondary mutations, such as C1156Y, L1171T, L1152R and L1198P promote ATP binding and stabilize the *ALK* active conformation [29,30] (Figure 1). These resistance mechanisms occur even more frequently after treatment with second-generation ALK inhibitors such as ceritinib and alectinib, reflecting their greater potency and selectivity [31]. Although these mutations also induce resistance to both first- and second-generation *ALK* inhibitors [32], brigatinib, another second-generation ALK inhibitor, is mainly active against secondary resistance mutations, with the exception of the G1202R [33]. Subsequently, the third-generation *ALK* inhibitor lorlatinib was developed to overcome *ALK* secondary resistance mutations, including G1202R, and has a potential indication for use after failure of other ALK inhibitors [34]. The identification of different *ALK* mutations when progression occurs on TKI treatment is crucial to enable the right drug to be offered to the right patient at the right time. Within this context, the use of liquid biopsy to monitor the development of mutations has become necessary. 

### 2.3. Anti-ROS Proto-Oncogene 1

Chromosomal rearrangements involving the *ROS1* gene were first described in NSCLC in 2007 [35]. Subsequently, a frequency of 1–2% was documented in this type of cancer, with a prevalence in younger patients, never or light smokers and ADC histology [36]. *ROS1*-rearranged lung tumors are “addicted” to *ROS1* for growth and survival [35], leading to a pharmacological sensitivity of the tumor to *ROS1*-directed TKIs [36,37]. After preclinical studies showed that crizotinib potently inhibits *ROS1* [37], the phase I PROFILE 1001 study of crizotinib in ALK-translocated patients was amended to include *ROS1*-rearranged patients, reporting an objective response rate (ORR) and disease control rate (DCR) to crizotinib of 72% and 90%, respectively [37]. Following these results, crizotinib was fully approved by the Food and Drug Administration (FDA) in March 2016 for the treatment of advanced *ROS1*-rearranged NSCLC. However, as happens in EGFR-mutated and ALK-translocated patients, *ROS1*-rearranged tumors typically develop resistance mechanisms to crizotinib involving secondary mutations in the *ROS1* kinase domain (50–60% of cases) or “off target” alterations in parallel pathways [38,39].

The most frequent ROS1 resistance mutation is the G2032R, which causes steric hindrance to drug binding, whilst not altering the oncogenic kinase activity [40]. Other resistance mutations include D2033N, S1986Y/F, L2026M and L1951R. G2032R and L1951R have been shown to confer the highest level of crizotinib-resistant phenotype in vitro [41]. Less is known about “off target” mechanisms. Alterations of the KIT proto-oncogene receptor tyrosine kinase (KIT), the KRAS proto-oncogene GTPase (KRAS), *EGFR* and *BRAF* genes have also been reported [42,43,44]. Phenotypic changes, such as epithelial-to-mesenchymal transition (EMT), have also been described [39]. Several other agents have proven active in patients with *ROS1*-rearranged tumors and in those developing ROS1 resistance mutations. In particular, ceritinib and brigatinib have shown comparable activity against L2026M but not against G2032R, D2033N or L1951R [38]. Whilst lorlatinib has proven effective against different resistance mutations [45], its activity against G2032R is limited. Despite cabozantinib being one of the few agents with confirmed activity against the G2032R mutation, its high toxicity severely restricts its use in this setting [46].

## 3. Immune Checkpoint Inhibitors

The development of immune checkpoint inhibitors (ICIs) introduced a new era in the treatment of lung cancer. Two agents directed against the programmed cell death protein 1 (PD-1), nivolumab and pembrolizumab, and one agent directed against the programmed death-ligand 1 (PD-L1), atezolizumab, have been approved for second-or-more line treatment of advanced lung ADC (Figure 1), but generate a durable clinical response in only 30% of patients [47,48,49,50]. Pembrolizumab is also approved for the first-line treatment of patients with PD-L1 expression >50%, with a 45% response rate and a median progression-free survival (PFS) of 10.3 months (95% confidence interval [CI] 6.7-not reached) [51]. In addition, recent evidence has highlighted the efficacy of a combination of chemotherapy (CT) and ICIs in the first-line setting, with a median PFS of 8.8 months (95% CI 7.6 to 9.2), independently of PD-L1 expression [52]. There are still no accurate predictive biomarkers to select patients who are more likely to respond to ICI therapy. PD-L1 expression is currently the only biomarker used but is unreliable as PD-L1-negative patients sometimes respond to therapy [47,48,49,50]. Although tumor mutational burden (TMB) represents another parameter capable of identifying patients who are more likely to respond to ICIs [53], a fully standardized method for its detection has not yet been established [54]. Liquid biopsy could potentially be of help in identifying biomarkers of resistance to ICI treatment during the course of treatment, thus bypassing the problem of tumor heterogeneity. However, there are numerous difficulties involved in developing targeted sequencing panels to for the assessment of TMB starting from cfDNA, and further research is needed to validate this approach [54].

Numerous studies have also reported a correlation between neutrophil-to-lymphocyte ratio (NLR) and immunotherapy [55,56,57]. In a recent report, Jiang et al. performed a comprehensive online search to explore the relation between blood NLR and OS and PFS in NSCLC patients treated with ICIs. They considered 16 studies, and found a significant association between elevated NLR values and patients’ prognosis, with shorter PFS and OS in those patients treated with ICIs and with elevated blood NLR [58]. 

An important role has also been reported in determining ICIs resistance by tumor associated macrophages (TAMs). In particular, Muraoka et al. demonstrated that in immune-resistant tumors, TAMs remained inactive and did not serve as antigen-presenting cells, and their manipulation, by inducing their antigen-presenting activity, could re-sensitize cells to treatment [59]. Moreover, TAMs enhance tumor hypoxia and aerobic glycolysis, contributing to resistance to therapy. Depletion of TAMs restores drug sensitivity and increases PD-L1 expression in aerobic cancer cells as well as T-cell infiltration in tumors, resulting in antitumor efficacy by anti-PD-L1 treatment [60].

## 4. Liquid Biopsy in Monitoring Response to Treatment

**Figure 2:**
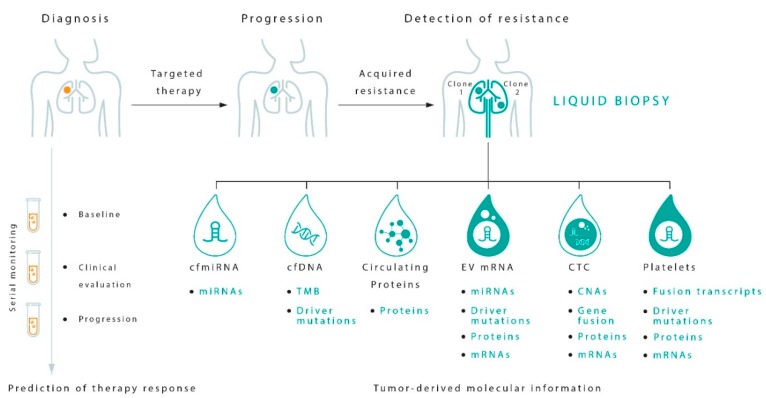
Potentials of tumor circulating biomarkers for prediction and monitoring response to therapy in NSCLC by liquid biopsy. TMB, tumor mutational burden; miRNA, microRNA; mRNA, messenger RNA; CNAs, genomic copy number alterations. A range of tumor components can be isolated from the blood of patients with NSCLC, such as cell-free DNA (cfDNA) and cell-free microRNAs (cfmiRNAs), circulating tumor cells (CTCs), extracellular vesicles (EVs), platelets and proteins released in the circulation. All these circulating cancer biomarkers are most suitable for different diagnostic applications to derive specific clinically relevant molecular information that may help predict the risk of relapse and the development of resistance mechanisms. While sequencing of cfDNA is the gold standard to determine the mutational status of the tumor, new technologies have enabled the advantage to be taken of CTCs, obtaining a complete and complementary view of tumor biology. EVs are a reservoir of miRNAs, and can integrate the information derived from cfmiRNAs. Platelets can often contain larger oncogenic transcripts and gene-fusion mRNAs. Analysis of circulating proteins may provide in a not-far future a wealth of information on tumor development. These methods pose liquid biopsy as a comprehensive diagnostic practice to yield the molecular heterogeneity of cancer. As a foreseeable application of liquid biopsy, serial blood testing holds great potential for minimally invasive dynamic monitoring of therapy efficacy, allowing early diagnosis of progression to metastasis.

The main use of liquid biopsy in NSCLC is that monitoring resistance mechanisms that develop with targeted agents or ICIs. Different types of samples are used for this purpose (Figure 2). The possibility of identifying resistance mechanisms during treatment could help to identify disease progression earlier and improve treatment personalization.

### 4.1. Cell Free DNA

#### 4.1.1. Cell Free DNA Biology

There is evidence that tumor cells release their DNA into the circulation [61]. The challenge in detecting cfDNA lies in the fact that cfDNA is markedly dilute compared to background circulating germline DNA (0.01–10%) [62]. This is one of the main reasons why highly sensitive methodologies are needed to determine cfDNA. A growing number of studies have investigated the usefulness of cfDNA in cancer management and, among these, the assessment of cfDNA for treatment monitoring and detection of resistance mechanisms is one of the most promising (Table 1).

#### 4.1.2. Clinical Application of cfDNA

A great deal of research has been performed to identify EGFR T790M mutations and monitor EGFR-sensitive mutations in cfDNA during the course of TKI treatment, using methodologies with different sensitivity [70,71]. These methods include real-time PCR-based approaches, digital droplet PCR, BEAMing and next generation sequencing (NGS) methodologies [72]. However, although all of these are high-sensitivity methodologies, sensitivity is fairly low (60–70%). Conversely, specificity is very high (90–100%) [10,73,74,75]. Hence, the absence of a specific mutation, as detected in cfDNA, does not exclude the possibility of that mutation being present in tumor tissue. Recent advances in NGS technology have demonstrated the possibility of obtaining high levels of both specificity and sensitivity [76,77], suggesting that the low sensitivity will probably be overcome. 

The guidelines from the College of American Pathologists, the International Association for the Study of Lung Cancer, and the Association for Molecular Pathology established that the use of cfDNA is indicated at diagnosis when tissue is limited and/or insufficient for molecular testing, and also for the monitoring of T790M mutation [78]. However, in either case, if the EGFR mutation test is negative in cfDNA, tissue biopsy should be considered. 

It was recently shown that NGS analysis of cfDNA in lung cancer with insufficient tumor samples for tissue sequencing could be extremely useful, facilitating the detection of actionable variants that frequently co-occur with other potentially clinically relevant genomic alterations and thus permitting the timely initiation of genotype-matched therapies [79].

Analysis of the T790M mutation should be always accompanied by evaluation of the sensitizing EGFR mutation to confirm that tumor DNA is being shed into the circulation. Oxnard et al. [80] reported a higher percentage of responders in patients with a double-negative cfDNA analysis (i.e., absence of T790M and EGFR-sensitivity mutation), than in those with negative T790M but positive EGFR-sensitivity mutation. This suggests that the absence of mutations in the former group of patients was mainly due to a “non-shedding” tumor, rather than to the actual absence of mutation. However, in both cases, tumor re-biopsy is recommended [72].

In addition to its proven usefulness in EGFR mutation analysis, cfDNA could also help to monitor secondary ALK resistance mutations during treatment with an anti-ALK agent [66,68,72,81]. As observed in patients progressing on EGFR-TKIs, those progressing during anti-ALK treatments may develop secondary ALK resistance mutations for which more specific anti-ALK agents might be effective. Given the wide range of mutations that need to be determined, NGS would seem to be the best methodology [72,76,77]. However, there is still insufficient evidence to support the use of testing ALK mutational status in lung ADC patients with sensitizing ALK translocation who have progressed after treatment with an ALK-targeted TKI [78]. Similarly, cfDNA analysis could potentially be used for ROS1-positive patients treated with crizotinib as they too may develop resistance mechanisms that could be used to drive subsequent targeted treatment [67,76,77]. However, there are still no clinical indications to indicate the ROS1 test in cfDNA.

With regard to ICIs, the only potentially detectable biomarker in cfDNA that could drive the choice of treatment is the TMB test. Gandara et al. demonstrated that it is possible to use cfDNA to determine TMB, also reporting a relationship between clinical outcome and cfDNA TMB [82]. However, further studies are needed to establish the conditions required for using this test in routine clinical practice, especially the choice of cut-off value to be used [54].

### 4.2. Circulating Tumor Cells

#### 4.2.1. Circulating Tumor Cell Biology

Although extremely rare, circulating tumor cells (CTCs) isolated from the peripheral blood of cancer patients represent a promising alternative to invasive biopsies as a source of tumor tissue for the detection, characterization, and monitoring of all non-hematologic cancers [69]. Most importantly, compared to single-site biopsy CTC analysis gives a comprehensive picture of the overall tumor content and of the intratumoral heterogeneity arising from branched clonal evolution [83,84]. The usefulness of CTC single-cell analysis lies mainly in its providing parallel information on the mutational profile of the tumor cell, copy number alteration (CNA), genomic rearrangement, and gene expression [85].

A crucial technical challenge to detecting CTCs stems from their rarity, e.g., one cell per milliliter of blood, among millions of background leukocytes [86]. To date, CTC-enrichment by immunomagnetic capture coupled with microfluidic devices has proven the most successful and most widely-used approach to isolate CTCs thanks to the presence of CTC-specific antigens and tumor markers of the tissue of origin that make them clearly distinguishable from leukocytes [87]. Downstream single-cell genetic and phenotypic profiling has made it possible to explore the mechanisms of metastasis and tumor-initiating capacity of CTCs [88]. Downregulation of the major histocompatibility complex (MHC) to avoid immune detection and survive the migration in the bloodstream [89], along with the expression of genes involved in EMT, would seem to be prerequisites of CTCs to initiate organ infiltration [90]. Detection of CTCs with EMT signature is associated with metastasis in NSCLC patients [91], where the presence of certain subpopulations of CTCs defines different tumor characteristics, and the total number of CTCs in the blood correlates with the onset of EGFR-mutated metastasis [92]. The phenotypic evolution of CTCs can be traced during treatment, revealing the appearance of stem cell features and resistance markers that denote the latency of CTCs in the circulation and their resistance to anticancer therapies [93].

#### 4.2.2. Potential Clinical Application of CTCs

In patients with lung cancer, complete CNA profiling of single CTCs generates a molecular classifier capable of predicting chemoresistance and clinical outcome [94]. Furthermore, combining genome-wide DNA methylation and RNA sequencing at single-cell resolution with functional drug screening assays has provided a useful insight into the association between CTCs and different types of drug resistance [95].

The clinical usefulness of CTCs in NSCLC has been further tested with various approaches in an attempt to raise the number of detectable CTCs per milliliter of patient’s blood and consequently increase the sensitivity threshold for the analysis of resistance mutations [69]. Using microfluidic CTC pre-enrichment followed by a PCR-based assay, it is possible to identify EGFR-mutation-positive patients by tissue biopsy with an accuracy of 94%. During anti-EGFR therapy, CTCs can also detect the emergence of the T790M resistance mutation [96]. However, cfDNA analysis has a clear advantage over CTCs in whole-exome sequencing for the detection of a broad range of resistance mutations to EGFR-directed therapy [93]. Pailler et al. detected EML4-ALK rearrangement in CTCs using size-exclusion microfiltration coupled with fluorescence in situ hybridization (FISH). The authors found a strong correlation between ALK-fusion status in CTCs and that of the tumor biopsy, detecting at least four ALK-rearranged CTCs per ml of blood in each patient tested. The CTCs expressed an homogenous pattern of ALK rearrangements, constituting in a unique population of cells with enhanced EMT phenotype [97]. Ilie et al. reported that ALK gene rearrangement carried by CTCs was confirmed by a strong ALK protein expression on the CTC surface and accompanied by the corresponding ALK rearrangement in the primary tumor [64]. Furthermore, real-time blood monitoring of ALK-rearranged CTCs during the course of therapy with crizotinib showed great promise in predicting the outcome of NSCLC patients with ALK-rearranged tumors [98].

It may be possible to evaluate resistance to immunotherapy in NSCLC by recovering CTCs that carry the PD-L1 protein on their plasma membrane. In a study by Ilié et al., the expression of PD-L1 on CTCs was isolated from advanced NSCLC patient blood samples, measured by immunocytochemistry (ICC) and matched with primary tumor staining, revealing 93% concordance between CTCs and tissue, and 55% specificity in detecting PD-L1-positive CTCs. Patients who had PD-L1-positive CTCs in circulation also had a trend for worse prognosis when undergoing chemotherapy [99]. Interestingly, the presentation of PD-L1 on CTC surface has been associated with disease progression and lack of response to anti-PD-1 therapy, which could reflect advanced disease status and immune escape mediated by other checkpoint pathways, probably activated by CTCs that have already begun transformation to an EMT-like phenotype [100]. These findings strongly suggest that PD-L1 measured in CTCs adequately reflects PD-L1 status in the primary tumor. However, the detection of PD-L1 protein in CTCs can also be used to select patients who are more likely to respond to anti-PD-1/PD-L1 treatment, as immunofluorescence has shown that PD-L1 is expressed, together with the epithelial cell adhesion molecule (EpCAM), in over 80% of CTCs derived from metastatic lung cancer [101]. Monitoring PD-L1-positive CTCs at baseline and during the course of therapy in NSCLC patients treated with nivolumab could thus help to predict clinical outcome.

Recent advances in isolating CTCs have enabled precise genetic analysis of drug-resistant CTC clones with specific signatures of direct clinical relevance. Such data support the notion that CTCs can simultaneously provide an overview of tumor genomics and gene expression, and that the characterization of CTCs may be a key factor as these cells are ultimately responsible for metastasis [90]. CTC counts work as independent prognostic factors at both baseline and during follow-up in patients with EGFR-mutated or ALK-rearranged NSCLCs undergoing standard chemotherapy or targeted therapies [102]. In patients with metastatic cancer receiving systemic treatment, temporal changes in the number of CTCs have been shown to correlate with clinical outcome, as measured by standard radiography [103]. Thus, the study of CTCs could have broad implications in elucidating the functional applicability of novel biomarkers, at the same time improving the clinical management of cancer. Nonetheless, CTC technology needs to be reliable, reproducible and robust, with clinical validation to ensure standardization of the readout. For this reason, the use of cfDNA remains preferable for mutational assays that seek to define biomarkers of immediate clinical utility. Table 2 summarizes the most relevant studies on detection of predictive biomarkers by the use of CTCs.

### 4.3. Extracellular Vesicles

#### 4.3.1. Extracellular Vesicle Biology

The discovery of the ubiquitous presence of a large number of extracellular vesicles (EVs) in the body fluids, coupled with the technical upgrades for EVs purification, is destined to make a substantial improvement in the detection of novel tumor biomarkers, from routine blood tests [104]. The investigation of the functional relevance of EVs flow into the circulation is ongoing, although their key role in cell-to-cell communication and in the transfer of biological material, typically functional microRNAs (miRNAs), messenger RNAs (mRNAs), or proteins, inside the recipient cells, is now widely accepted [105,106].

There is evidence that EVs may be directly involved in cancer resistance to therapy, and tumor EV-derived information could be used for the early diagnosis of the metastatic propensity of a primary tumor [107]. Tumor EVs can promote the organ-specific metastasization of the cancer cell type they have originated from [108], by preparing the pre-metastatic niche through the engagement of the signaling pathway of normal cells [109,110], while also allowing further uptake of malignant EVs [111]. For example, the conversion of quiescent cells to a pro-tumorigenic phenotype by tumor EVs could be stimulated by the transfer of cellular protein kinases, and the correlation between phosphorylation of protein kinases in EVs and that in the tumor tissue could serve as a predictive biomarker of tumor evolution during TKI treatment [112]. 

#### 4.3.2. Potential Clinical Application of EVs

Tumor EVs are capable of transferring the oncogenic ALK isoform from cancer cells, conferring drug resistance to sensitive cells, via activation of the MAPK pathway, which indicates that EVs could be a vehicle for the onset of therapeutic resistance [113]. Likewise, NSCLC cells can alter the function of adjacent cells by the exchange of EVs that transport active EGFR [114,115]. Interestingly, nuclear translocation of EGFR seems to be mainly dependent on EV fusion mechanism, rather than specific nuclear localization signals [116]: once in the nucleus, EGFR activates distinct signaling pathways that are associated with tumor resistance to therapy-induced DNA damage and anti-EGFR treatment [117,118]. Detection of circulating EGFR protein has long been envisaged as an important prognostic marker in many cancers, including NSCLC [119], and now this information might finally be complemented with a more accurate analysis of EV protein content, to achieve the best prediction to anti-EGFR therapy [120]. EVs can also transport nucleic acids molecules of wild-type *EGFR* and mutated *EGFR* reflecting the genetic signature of the original tumor [121]. In a recent study by Hur et al. EVs isolated from plasma and bronchoalveolar lavage fluid (BALF) of NSCLC patients were used to genotype the *EGFR* status via liquid biopsy, with high accuracy, demonstrating improved specificity and sensitivity, compared to cfDNA analysis [122]. The DNA of *EGFR*, extracted from both plasma and BALF EVs, contained the L858R mutation and the exon 19 deletion, showing 100% accordance with tissue biopsy, where sensitivity of cfDNA was around 70%. EV liquid biopsy also enabled the authors to detect the T790M mutation with high sensitivity, later confirmed by tissue re-biopsy, in patients who developed resistance to TKIs [122].

In addition to influencing the phenotype of adjacent cells by sharing EV cargo and preparing the pre-metastatic niche in distant sites, cancer cells can also release a continuous amount of immunomodulatory EVs [123] that increase substantially during cytotoxic treatment [63]. The fact that EVs modulate immune cells has been known for a long time [124], as they are a central element in the antigen presentation process, via both the MHC classes I and II [125], and in the receptor–ligand interaction via the tetraspanin network [106]. At the same time, tumor-derived EVs may be the conduit for immune surveillance escape by cancer cells, leading to the failure of immunotherapies [126]. Tumor EVs probably succeed in deceiving lymphocyte activation through the exposure of inhibitory ligands that elicit the immune checkpoint response. Upregulation of PD-L1 expression on the plasma membrane is indeed a common adaptation used by cancer cells [127], with levels of circulating PD-L1 clearly correlating with tumor aggressiveness [128]. PD-L1 positive EVs inhibit CD8+ T cell activation, and their presence in the blood of patients with metastatic cancer identifies those who are more likely to respond to anti-PD-1 therapy [129]. The positive response to anti-PD-1 antibodies can also be predicted by measuring the expression PD-L1 mRNA in the EVs of NSCLC patients [130].

Mechanisms of acquired resistance to ICI treatments can emerge at different molecular levels, and tumor-derived EVs have been shown to play a crucial part in this process [131]. Tumor EVs impair T cell function and cytotoxic activity of natural killer (NK) cells by releasing TGF-β into the microenvironment [132] or by delivering miRNAs into T cells, which results it the downregulation of genes required for the activation of the inflammatory process [65]. Tumor EVs also substantially reduce proliferation of CD8+ T cells and NK cells [133]. They contribute to a tumor suppressive microenvironment by delivering a non-coding RNA that induces the expression of PD-L1 on antigen-presenting cells, also stimulating concurrent cancer-promoting inflammation by the release of cytokines by monocytes [134]. 

Monitoring the presence of tumor-derived EVs, by blood tests and the investigation of the vesicle-specific content could prove to be crucial for rapid and accurate diagnosis or for early tumor detection [135]. Parallel screening for antigenic EV and analysis of EV biomarkers during the course of follow-up could help to define the best personalized treatment and also predict resistance. Although key technical and practical issues result mainly in the collection of heterogeneous populations of vesicles of unknown origin [136], limiting the use of the EVs in routine clinical practice, emerging technologies will probably overcome these limitations in the not-too-far future [135,137]. Table 3 summarizes the most relevant studies on detection of predictive biomarkers by the use of EVs.

### 4.4. Circulating miRNA

#### 4.4.1. Circulating miRNA Biology

Extracellular miRNAs are detected in all body fluids and account for over 50% of all the RNA species in the blood plasma [138]. These molecules are major modulator of gene expression, by guiding the degradation and translational repression of specific mRNA targets [139]. Most of the circulating cell-free miRNAs (cfmiRNAs) involved in cell-to-cell communication are selectively delivered to target cells by loading them into EVs, although functional miRNAs may also be released into the blood circulation by dying cells [140]. These cfmiRNAs have proven their diagnostic and prognostic relevance, in NSCLC, as in many other solid tumors [140].

In the course of the past ten years a multitude of alternative approaches have been used for cfmiRNAs extraction from blood, with a different impact on the quality of the results and the sensitivity of target detection. However, none have been designated as the effective standard procedure [141]. The most widely used techniques to analyze blood-recovered miRNAs are quantitative PCR, microarrays and deep sequencing (miRNA-seq), the last one having consistently improved the characterization of miRNA profiles of patients [140]. Expression profiling of total blood miRNAs can help the detection of early stage tumors [142] and could provide useful prognostic and predictive information [143,144]. For example, detection of circulating miR-21 and miR-10b, two of the most widely investigated cfmiRNAs, facilitates the diagnosis of lung cancer [145]. miR-21, targeting the *phosphatase and tensin homolog* (*PTEN*) gene, among others, is substantially overexpressed in patients with advanced-stage diseases and poor survival [146], while high concentrations of miR-10b can also correlate with disease progression and metastasis in NSCLC [147]. 

#### 4.4.2. Potential for Use of miRNAs in Clinical Practice

Circulating miRNAs have an important role in the development of drug resistance in many cancers, and can be used as efficient biomarkers to predict therapeutic response to TKIs in NSCLC [148]. For examples, blood levels of miR-21, increases from baseline to the time of acquired resistance in patients treated with first-line anti-EGFR TKIs [149]. Li et al. also found that miR-21 levels were higher in lung cancer cells with acquired resistance to TKIs than in TKI-sensitive cells. By deregulating the expression of *PTEN* and the *programmed cell death 4* (*PDCD4*), miR-21 could induced TKI-resistance via activation of the AKT pathway. These cells had no T790M mutations or *MET* alterations, suggesting that the upregulation of miR-21 could represent a new mechanism of acquired resistance in NSCLC, as confirmed also in animal models [149]. In a cohort of 105 non-smoker patients with lung ADC, circulating miR-195 and miR-122 expression levels were found to be associated with mutant-*EGFR* tumors and were also independent predictors of survival [150]. Elevated levels of miR-127 promote shift to EMT in lung cancer cells treated with anti-EGFR TKIs: in a regulatory loop involving the inflammatory signals of the nuclear factor kappa-light-chain-enhancer of activated B cells (NF-κB) downstream to EGFR, miR-127 was found as a self-reinforcing factor capable of sustaining the oncogenic transition [151]. Kwok et al. assayed serum EV-RNA serially by quantitative PCR, and revealed that circulating miR-21-5p and miR-486-3p transported by EVs correlate with disease progression in *EML4-ALK*-translocated NSCLC patients treated with anti-ALK agents. These two miRNAs were differentially expressed by EVs secreted by resistant subclones, which in turn induced drug resistance in recipient cell [152].

Numerous miRNAs transferred via EVs have a role in anti-tumor immunity [153], and can exert key immune-modulatory and pro-oncogenic functions by targeting a multitude of NK and T cell receptor mRNAs [154], or downregulating the expression of genes involved in the inflammatory response [65]. However, miRNAs transported by EVs, like those secreted by lung cancer cells in hypoxic conditions, can induce tumor infiltration of pro-oncogenic macrophages via transfer of miR-103a, and subsequent reduction of PTEN levels [155]. Recently, characteristic EV-miRNA signatures of cancer-related inflammation have been associated with prediction of response to anti PD-1/PD-L1 therapy in NSCLC [156]. Finally, the relative enrichment of certain miRNAs can be specific for each EV subtype, classified on the basis of the presence of characteristic surface markers, thus defining a functional signature of cancer EV-miRNAs that could enable improved screening for drug resistance [157].

Although much of the research carried out to date on circulating RNA remains exploratory, blood-based RNA profiling of cancer patients provides a valuable source of information to improve our understanding of tumor evolution and its adaptation to therapeutic pressure in clinical studies [158].

### 4.5. Platelets

#### 4.5.1. Platelet Biology and Involvement in Cancer

Analysis of platelet RNA content has recently emerged as an alternative, highly sensitive and accurate method for blood-based diagnostics in cancer. Platelets are anucleate blood cells deriving from megakaryocytes after a cellular redistribution of organelles and vesicular structures; they have multiple functions and very short lifespan [159]. Hence, although platelets do not contain their own RNAs, they are capable of capturing those RNAs that are released into the circulation by other cells, through both EV-dependent and -independent mechanisms. Platelets are highly receptive cells that can adapt easily to the environment, and they are very active in secreting regulatory factors and EVs [159]. By virtue of these properties, platelets are thought to be a major contributor to cancer progression and metastasization, not only because of their role in the coagulation process, but also because they create a microenvironment that protects tumor cells from immune elimination and facilitate their extravasation into the connective tissues [160]. Cancer cells can prepare their niche by secreting EVs that will transfer oncogenic RNAs into circulating platelets, which could then release these RNAs at the metastatic site [161]. These so-called tumor-educated platelets change their RNA expression profile in response to contact with tumor cells, reflecting the molecular signature of the tumor tissue, with important diagnostic consequences. Best et al. reported that the sequencing of platelet-associated RNA yielded about 5000 differently expressed RNAs between cancer patients and healthy controls, with an overall sensitivity of 97%, a specificity of 94% and an accuracy of 95% [162]. This analysis discriminated NSCLC patients with localized and metastasized tumors from healthy individuals with 75% accuracy, providing useful information about the primary tumor. The authors also accurately identified mutated *EGFR*, *KRAS*, and amplified *MET* tumors, with a specificity of 81%, 85% and 75%, respectively, and with a sensitivity of 90%, 94% and 100%, respectively [162]. 

#### 4.5.2. Clinical Potentiality of Platelets in NSCLC

In NSCLC, platelets are associated with resistance to TKI therapy, as they can also transport growth factors and cytokines that induce a bypass of EGFR signaling [163], while the number of blood platelets is, in itself, an independent predictor of survival for patients having *EGFR*-mutant tumors that are treated with anti-EGFR TKIs [164]. Analysis of blood platelets can also predict resistance to crizotinib and PFS of NSCLC patients with *EML4-ALK* rearrangements [165]. Transcripts of *EML4-ALK* are transferred into blood platelets by EVs released from cancer cells. Nilsson et al. detected *EML4-ALK*-translocated RNA in platelets can be achieved with 65% sensitivity and 100% specificity, permitting the monitoring of response to crizotinib and defining patient outcome. By measuring *EML4-ALK* transcript in platelets, crizotinib resistance was discovered two months before the radiographic detection of disease progression [165]. Table 4 reports the main studies that analyze platelets biomarkers.

Despite the risk of false positive and the possibility of non-cancer-related systemic factors affecting results, the analysis of platelets RNA content shows promise for improving current NSCLC diagnostic tests, and for predicting the course of disease during the development of drug resistance mutations. Given that platelets are, per se, huge collectors of EV-derived RNA, the detection of tumor-derived RNA in platelets could help to bypass the problems of the rapid degradation of circulating RNAs or low number of EVs containing mutated or rearranged transcripts. 

### 4.6. Circulating Proteins

Secreted proteins from cancer cells have a key role in the oncogenic process, while serving as useful tumor biomarkers. Hence, a great effort has been carried out in the recent years to define the cancer secretome [166]. Proteomic profiling of cancer tissue and liquid biopsy, coupled with mutational cfDNA analysis, incorporated into a logistic regression algorithm, has been successfully applied to track lung cancer development and gain information on early disease detection [167]. So far, however, large-scale approaches in NSCLC have been confined to tissue analysis and cell lines, and there is a paucity of information about the correlation between clinical response and the levels of circulating protein markers [168,169]. Zhang and colleagues used proteomics of lung cancer cells and tissues from in vivo models to define a detailed map of post-transcriptional modifications of receptor proteins and downstream kinase pathways, modulated by afatinib and erlotinib, and found that quantification of chances in major sites of phosphorylation in mutant EGFR signaling could predict the degree of response to TKI treatment [170]. It would be interesting if this information could be translated to circulating proteins. However, the identification of relevant protein markers in liquid biopsy is, at the moment, further limited by a lack of the adequate sensitivity and specificity that are required for an optimal clinical biomarker [171]. Initially, quantitative mass spectrometry helped identify differentially expressed proteins in body fluids and predict clinical outcome during chemotherapy or radiotherapy [172,173]. Proteomic analysis extended to serum samples of NSCLC patients treated with erlotinib demonstrated that the presence of distinct subsets of protein biomarkers are associated with OS and PFS [174]. Most relevant predictive protein biomarkers were components of the MET pathway, including the hepatocyte growth factor (HGF), and pro-inflammatory cytokines. Growth factors and cytokines were indeed among the first serum biomarkers being studied by traditional methods, like enzyme-linked immunosorbent assay (ELISA), in association with NSCLC therapeutic resistance [175]. For example, dynamic evaluation of the serum levels of HGF, as wells as those of interleukin-6 and vascular endothelial growth factor (VEGF), during the course of therapy, could be used to predict the efficacy of anti-EGFR-TKI treatment in NSCLC patients with mutant *EGFR* [176,177]. The predictive value of serum proteins emerged also in the evaluation of the clinical response of NSCLC patients to nivolumab: changes in the levels of several cytokines, chemokines, growth factors and angiogenesis factor, from baseline to the first clinical evaluation, showed clear association with the outcome [178].

It is likely that proteins found in circulation are not always free, but transported by extracellular structures, or are part of CTC content. As discussed above, proteins that can have clinical relevance for prediction of therapy response in NSCLC, like mutant EGFR and ALK, or PD-L1, are frequently found associated with EVs [179]. Proteomic approaches have shown a differential abundance in protein content between normal and lung cancer-derived EVs, with an enrichment for proteins involved in signal transduction, including EGFR [180]. In future, combined proteomics of tissue and body fluids with improved bioinformatics analysis may help overcome the limitation of this approach in sensitivity for the use in routine liquid biopsy.

## 5. Conclusions

Since cfDNA-based assays are relatively low in complexity, in terms of purification and analysis, they are currently used in the clinical practice of NSCLC, and are considered the gold standard for defining the tumor mutational status and predicting treatment response by liquid biopsy. Analysis of CTCs can provide, however, an unparalleled mean to interrogate tumor biology and metastatic evolution. At the same time, the development of more reliable and low-cost technologies for the isolation of EVs and purification of their nucleic acid content makes probably EV analysis the new frontier of cancer diagnostics. Biologically speaking, EV release represent an active and most pliant mechanism that tumor cell can use to influence the surrounding microenvironment and communicate with distant sites, preparing the metastatic niche and shutting down the immune response. Secondly, investigating EV contents and surface markers that can track back to the cell of origin of primary tumors would make diagnosis and prediction of therapy response more precise and accurate, given the relative abundance of tumor-derived EVs in circulation compared to CTCs. On the other side, release of cfmiRNAs may account for a specific and more dynamic mechanism of resistance adopted by cancer cells, therefore it can bring up unique information of disease progression. Recent advances in proteomics have provided novel means for adding circulating proteins to the arsenal of the liquid biopsy biomarkers. So far, proteomics has provided a wealth of information from tissue specimen, but its potential use for blood-based tests is limited by the insufficiency in sensitivity and specificity needed of the clinics. It is quite evidently tough that each biomarker comes with its own limitations. Potentially, it would be the complementation of all different methodologies of analysis to provide the most accurate prediction of therapy response and define the best biomarker utility, depending on tumor stage and patient’s condition. Although at the moment only *EGFR* mutation analysis on cfDNA is used and has a role in the clinical practice, other determinations will probably have a clinical impact on treatment decision making in the near future, such as *ALK* and *ROS1* mutation analyses, especially during the course of therapy to monitor the onset of resistance mechanisms. Moreover, while mutation analysis based on cfDNA is the easier and cheaper approach in liquid biopsy, other biological sources (CTC, EVs and platelets) might better perform in the detection of gene fusions and genomic rearrangements. However, the issue remains that all current methodologies must be improved and standardized for their potential usefulness in the clinical practice.

## Figures and Tables

**Table 1:** Principal resistance biomarkers detectable by cell free DNA (cfDNA).

Biomarkers	*N* Patients	Methodology	Sensitivity (%)	Specificity (%)	Reference
EGFR T790M	23	PNAClamp™	71.4	100	Hur et al. [63]
	21	SARMS	39	100	Maheswaran et al. [64]
	208	ddPCR™	-	57	Jenkins et al. [65]
	226	cobas^®^	-	51	Jenkins et al. [65]
	227	NGS	-	65	Jenkins et al. [65]
	216	BEAMing	69	70	Oxnard et al. [66]
ALK resistance mutations	7	ddPCR™	2 mutations detected	-	Yoshida et al. [67]
	20	ddPCR™	10 mutations detected	-	Bordi P et al. [68]
ROS1 resistance mutations	18	NGS	6 mutations detected	-	Dagogo et al. [69]

Abbreviations: PNAClamp™, peptide nucleic acid (PNA)-mediated PCR-EGFR Mutation Detection Kit; SARMS, Scorpion Amplification Refractory Mutation System; ddPCR™, Droplet Digital ™ PCR; cobas^®^, cobas^®^ EGFR Mutation Test v2; NGS, next generation sequencing; BEAMing, Beads Emulsion Amplification Magnetic digital PCR.

**Table 2:** Principal clinical biomarkers detectable by circulating tumor cells (CTCs).

Biomarkers	*N* Patients	Methodology	Sensitivity (%)	Specificity (%)	Reference
EGFR T790M	21	SARMS	94	100	Maheswaran et al. [64]
EML4-ALK	39	FISH	100	100	Pailler et al. [100]
	32	FA-FISH	100	100	Pailler et al. [98]
	87	FISH	100	100	Ilie et al. [99]
PD-L1 expression	71	ICC	55	100	Ilie et al. [101]

Abbreviations: SARMS, Scorpion Amplification Refractory Mutation System; FISH, fluorescence in situ hybridization; FA-FISH, filter-adapted fluorescence in situ hybridization; ICC, immunocytochemistry.

**Table 3:** Principal clinical biomarkers detectable by extracellular vesicles (EVs).

Biomarkers	*N* Patients	Methodology	Sensitivity (%)	Specificity (%)	Reference
EGFR T790M	23	PNAClamp™	100	100	Hur et al. [63]
PD-L1 expression	8	ddPCR™	-	-	Del Re et al. [132]

Abbreviations: PNAClamp™, peptide nucleic acid (PNA)-mediated PCR-EGFR Mutation Detection Kit; ddPCR™, Droplet Digital ™ PCR.

**Table 4:** Principal clinical biomarkers detectable by platelets.

Biomarkers	*N* Patients	Methodology	Sensitivity (%)	Specificity (%)	Reference
EGFR T790M	283	NGS	96	92	Best et al. [164]
EML4-ALK	77	RT-PCR	65	100	Nilsson et al. [163]

Abbreviations: NGS, next generation sequencing; RT-PCR, reverse transcription PCR.
